# Haploinsufficiency of *Whrn* Contributes to Progressive Sensorineural Hearing Loss in C57BL6 Mice

**DOI:** 10.1007/s10162-025-00991-4

**Published:** 2025-05-13

**Authors:** Han-Gyu Bae, Sean Kashiwagura, Andrew Jung, Elizabeth Gould, Jun Hee Kim

**Affiliations:** 1 Department of Otolaryngology Head and Neck Surgery, Kresge Hearing Research Institute, University of Michigan, Ann Arbor, MI USA; 2 Department of Cell and Developmental Biology, University of Michigan, Ann Arbor, MI USA; 3 Department of Cellular and Integrative Physiology, University of Texas Health Science Center, San Antonio, USA

**Keywords:** Whirlin, Hearing loss, Aging, Sex, Usher syndrome type 2

## Abstract

**Purpose:**

*Whrn*, encoding whirlin, is one of the genes highly relevant to Usher syndrome (USH) that has been known as an autosomal recessive genetic disorder that is characterized with sensorineural hearing loss with retinitis pigmentosa. Although recent studies on the other USH genes, *PDZD7* and *Ush1 g*, showed a possibility of haploinsufficiency effect, the potential contribution of heterozygous *Whrn* loss to hearing loss remains unclear.

**Methods:**

To investigate the effect of *Whrn* haploinsufficiency, we conducted a longitudinal study assessing auditory function in heterozygous *Whrn* mutant (*Whrn*^+*/−*^) mice in which long isoform of *Whrn* was deleted by replacing exon 1 with Neo cassette without disturbing short isoform. The threshold of auditory brainstem responses (ABRs) was measured on 135 *Whrn*^+/*−*^ mice and littermate 133 wild-type (WT) mice from 1 to 6 months of ages. From those data, the threshold from male and female were separately analyzed to investigate sex-dependent effect. To further investigate underlie mechanisms, hair cell death was investigated using immunohistostaining from 4 to 5 WT, 5 female *Whrn*^+*/−*^, and 7 male *Whrn*^+*/−*^ mice at 4–5 months old.

**Results:**

Hearing threshold was significantly increased with aging in *Whrn*^+*/–*^ mice compared to WT controls. Both WT and *Whrn*^+*/–*^ mice exhibited sex-dependent variations in hearing sensitivity. Notably, *Whrn*^+*/–*^ males showed a progressive hearing loss with age, while *Whrn*^+*/–*^ females exhibited elevated hearing thresholds as early as 1–2 month of age compared to WT females.

**Conclusion:**

These results provide evidence for a haploinsufficiency effect of *Whrn* on auditory function and highlight its potential role in progressive sensorineural hearing loss.

## Introduction

Usher syndrome (USH) is an incurable genetic disease characterized by combined sensorineural hearing loss, vison loss due to retinitis pigmentosa (RP), and vestibular dysfunction, affecting 1.6–4.4 per 10,000 individuals worldwide [1, 2]. USH symptoms are heterogeneous from moderate to severe depending on the genetic makeup of the individual [3]. To date, 10 causative genes have been identified for USH, with *MYO7A* accounting for > 50% of type 1 USH and *USH2A* contributing to approximately 80% of type 2 USH [3]. Although USH is well described as an autosomal recessive disorder, a synergistic effect by the combination of heterozygous mutations in USH genes has been identified [4]. It suggests the potential contribution of heterozygous mutations on those genes to the phenotypes of USH. However, the haploinsufficiency effect of USH genes and the genotype–phenotype relationships of USH genes on hearing loss remain to be explored.

lWhirlin, encoded by *Whrn*, is a PDZ domain-containing cytoskeletal scaffold protein localized at the tip of the stereocilia and plays a role in stereocilia elongation and maintenance in both inner and outer hair cells [5–7]. Mutations in *Whrn* cause impairments in hearing, balancing, and vision as observed in USH type 2 (USH2), which is the most prevalent classification of USH. Since USH2 has been described as an autosomal recessive genetic disorder, the carriers with a single copy of the mutation (heterozygous) are considered to have normal hearing without symptoms, which leads a lack of studies on heterozygous carriers with most studies for USH2 on homozygous mutant models. Recent studies demonstrated that heterozygous mutations in other USH associated genes (e.g., *PDZD7*) act synergically with other genes and have a compound effect on pathological phenotypes in patients [4, 8]. A patient with a heterozygous frameshift in *PDZD7* (another USH2 gene that have high similarity of structure with *Whrn*) had an earlier onset and more severe retinitis pigmentosa than a sibling without the mutation, suggesting the potential haploinsufficiency effect of USH genes on hearing loss [4, 9]. However, the effect of heterozygous *Whrn* mutations in USH mouse models on sensorineural hearing loss have not been evaluated. Here, we investigated the how *Whrn* haploinsufficiency exacerbates hearing impairment using the mice that is C57BL6/J background with homozygous *Chd23*^ahl^ allele and heterozygous deletion of *Whrn* long isoform.

## Materials and Methods

### Ethics Approval

All the animal experiments followed ARRIVE guidelines and conducted under the protocols approved by the University of Texas Health Science Center San Antonio (UTHSCSA) Institutional Animal Care and Use Committee (#20140045 AR).

### Animals

To investigate the haploinsufficiency of *Whrn*, mice with heterozygous *Whrn* mutant (MGI:4462398, *Whrn*^tm1 Tili^, C57BL6/J background with homozygous *Chd23*^ahl^ allele, here after *Whrn*^+*/–*^) in which long isoform of *Whrn* was removed by replacing exon 1 with Neo cassette without disturbing short isoform and its littermate WT (*Whrn*^+*/*+^) were used as a control. To investigate age-dependent hearing loss, from 1 to 6 months old mice, that the different age groups were from different batch, were used. The mice were kept on a 12:12-h light cycle with ad libitum food and water access.

### Auditory Brainstem Responses (ABRs)

To test ABRs, mice were anesthetized with 3.7% isoflurane and maintained with 2.3% isoflurane during recording (1 L/min O_2_ flow rate). ABR recordings were performed in a sound attenuation chamber (Med Associates, Albans, VT). Active, reference, and ground electrode (Rochester Electro-Medical, Lutz, FL) were placed in the subdermal space of the head, ipsilateral mastoid, and contralateral mastoid, respectively. Acoustic stimuli were generated by an Auditory Evoked Potentials Workstation (Tucker-Davis Technologies, Alachua, FL). Closed-field click stimuli which consisted of a series of amplitude-modulated square waves (0.1 ms of duration) were provided to the left ear through a 10 cm plastic tube (Tygon; 3.2 mm outer diameter) connected to TDT Multi-Field Magnetic Speakers. Sound stimulation intensities ranged from 90 to 10 dB SPL, with 5 dB SPL decrements, and a total of 512 recording (16 Hz of sampling rate) from each stimulation were averaged. Tone tests were performed at 8, 12, 16, and 32 kHz closed field stimuli with various stimuli intensity ranging from 90 to 20 dB SPL with 10 dB SPL decrements. The sampling rate for tone tests was the same as those from click test.

### Cochlear Tissue Preparation

Following the decapitation of the mouse, the cochlea was quickly removed and placed in a solution of 4% paraformaldehyde (PFA) in phosphate-buffered saline (PBS). A small hole was made at the apex of the cochlea and 4% PFA was perfused through the oval window and incubated in 4% PFA overnight for further fixation. Before dissection, the cochleae were decalcified with 10% EDTA for a week. The 10% (w/v) EDTA solution was switched every 24 h until the cochleae were soft enough to dissect. Then, the cochleae were dissected into the three turns: basal, middle, and apical regions for tonotopic differentiation.

### Immunohistochemistry

The dissected tissues were incubated with the Myo7a (Rabbit Igg, Proteus, Cat. #25–6790) antibody overnight at 4 °C followed by the secondary antibody (Anti-rabbit Igg Alexa Fluor™ 594, Invitrogen, Cat. #A-11012) treatment at a 1:500 dilution at 4 °C overnight. The tissues were washed with PBS three times between antibody treatment and before mounting. The mounted tissues were imaged using confocal microscopy (LSM-710; Zeiss).

### Statistical Analysis

Experimental data were analyzed and presented using Prism (GraphPad Software, San Diego, CA) and R with customized script. For statistical significance of multiple variables for ABR threshold and DPOAE, we used 2-way ANOVA test with multiple comparison followed by Šidák correction. The Kruskal–Wallis test with multiple comparison followed by Šidák correction for comparing hair cell death. For comparing change of patterns, we used the linear regression test. Data collected as raw values are shown as mean ± s.e.m. Details of statistical methods are reported in each figure legend. For all analyses, *p*-values < 0.05 were considered significant.

## Results

### Age-Dependent Hearing Impairment upon the Haploinsufficiency of Whrn

**Fig. 1:**
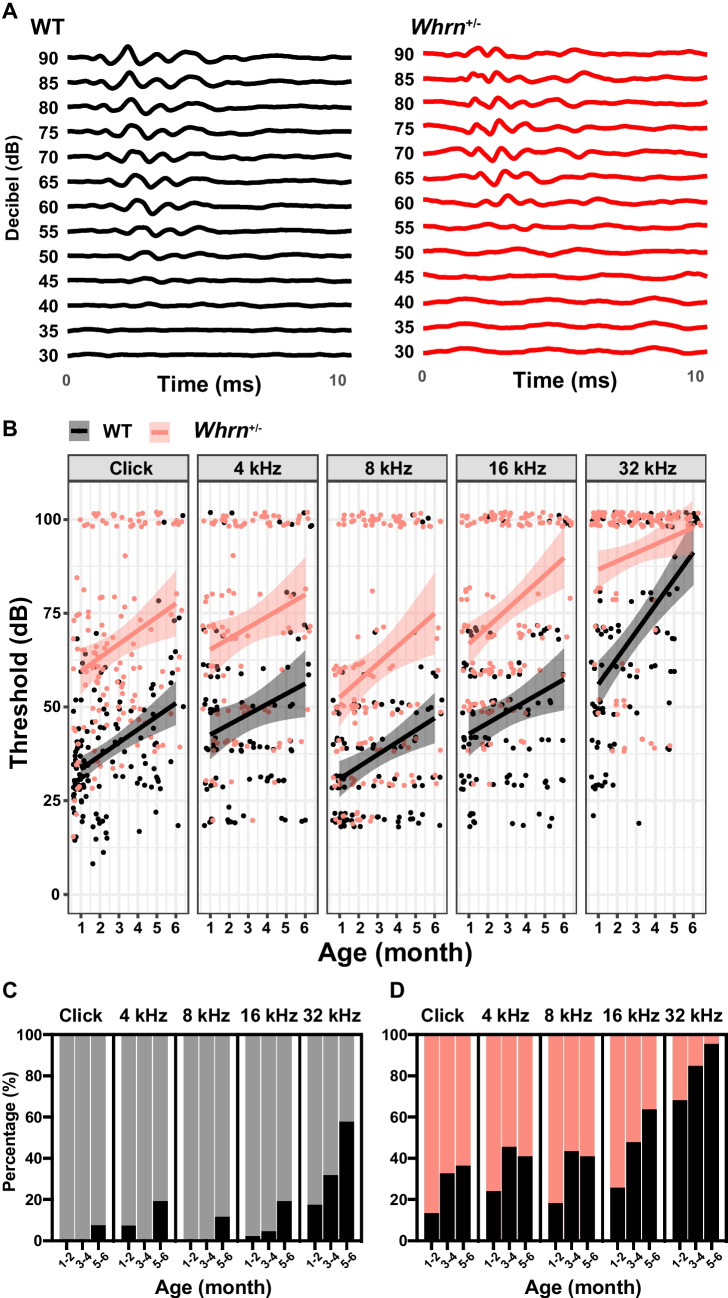
Age-dependent hearing impairment *Whrn* heterozygous mice. **A** Representative traces of ABRs in response to click stimuli (90 to 30 dB SPL) from WT and *Whrn*^+*/–*^ mice at 2 months old. **B** Summary of ABR threshold in response to click and tone stimuli (click, 4, 8, 16, and 32 kHz) in WT (black dots, *n* = 133 across 1–6 months age) and *Whrn*^+*/–*^ mice (red dots, *n* = 135 across 1–6 months age). The linear regression was conducted at each group and were presented with 95% CI (black and red lines). **C**, **D** The proportion of deaf mice (not detecting any signal at 90 dB) among total recorded WT (**C**) and *Whrn*^+*/–*^ mice (**D**) at 1–2 months, 3–4 months, 5–6 months old. Black bar indicates deaf mice portion, and those were presented with percentage

To investigate the impact of the haploinsufficiency of *Whrn* on hearing sensitivity throughout adulthood, mice with heterozygous deletion of long isoform of whirlin (*Whrn*^+*/–*^) were used. Using ABR recordings, we evaluated auditory transmission along the auditory pathway in *Whrn*^+/-^ mice at different ages (from 1 to 6 months old). The ABRs using various sound stimuli (click, 4, 8, 16, and 32 kHz) were measured from WT and *Whrn*^+*/–*^ mice. The intensity of sound stimuli (from 90 to 10 dB SPL for click stimuli and from 90 to 20 dB SPL for 4, 8, 16, and 32 kHz tone stimuli) at which discernable signal was no longer present was considered as a threshold (Fig. 1A). The threshold of individuals that had no detectable response at 90 decibels (dB SPL) were given a value of 100 dB SPL, and it was considered deaf. Although both WT and *Whrn*^+*/–*^ mice have elevated thresholds across all frequencies along with ages from 1 to 6 months, *Whrn*^+*/–*^ mice showed a stronger pattern of hearing impairment (Fig. 1B). In WT mice, the threshold at click stimuli was 34.83 ± 1.43 dB SPL (*n* = 59) at 1 month old and gradually increased to 60.00 ± 7.03 dB (*n* = 10) at 6 months old. In *Whrn*^+/*–*^ mice, the threshold was 59.14 ± 4.47 dB SPL (*n* = 29) at 1 month old and reached to 76.82 ± 6.51 (*n* = 11) at 6 months old. Similarly, higher thresholds were observed across various tone of stimuli in *Whrn*^+*/–*^ mice. The rate of age-related threshold increases was not significantly different between WT and *Whrn*^+*/–*^ mice at low frequencies (click, 4 to 16 kHz tone). Notably, the progression of high-frequency hearing loss was greater in *Whrn*^+*/–*^ mice at 32 kHz (*p*_*slope*_ = 0.01, linear regression).

While analyzing the threshold change of ABR, the proportion of mice without responding across all frequencies of sound stimuli was gradually increased in *Whrn*^+*/–*^ mice through ages. Therefore, considering the mice not responding to loud sound stimuli (90 dB SPL) as deaf, the proportion of deaf mice number was significantly increased in *Whrn*^+*/–*^ mice comparing to WT mice (Fig. 1C, D). A small proportion of WT mice showed deafness at 1–2 months old (*n* = 46–84, click: 0.00%, 4 kHz: 7.32%, 8 kHz: 0.00%, 16 kHz: 2.17%, 32 kHz: 17.39%) across all stimuli. Although the threshold of WT mice increased at 5–6 months old, the proportion of mice with hearing impairment remained low (*n* = 26–27, click: 7.41%, 4 kHz: 19.23%, 8 kHz: 11.54%, 16 kHz: 19.23%, 32 kHz: 57.69%). However, the proportions of deafness in *Whrn*^+*/−*^ mice were much higher than in WT even at 1–2 months old (*n* = 54–67, click: 13.43%, 4 kHz: 24.07%, 8 kHz: 18.18%, 16 kHz: 25.76%, 32 kHz: 68.18%) and distinctly increased with ages. At 5–6 months old, the hearing loss is more severe in *Whrn*^+*/−*^ mice than WT showing almost complete loss of high-frequency hearing (*n* = 22, click: 36.36%, 4 kHz: 40.91%, 8 kHz: 40.91%, 16 kHz: 63.64%, 32 kHz: 95.46%, Fig. 1C, D). Taken together, heterozygous *Whrn*^+*/−*^ mutant carriers showed hearing impairment with greater rate of deafness in early adulthood (at 1 month old) and significantly elevated thresholds of ABRs at all frequencies, indicating an early onset of age-related hearing loss.

### The Effect of *Whrn* Haploinsufficiency on Hearing in Sex-Dependent Manner

**Fig. 2:**
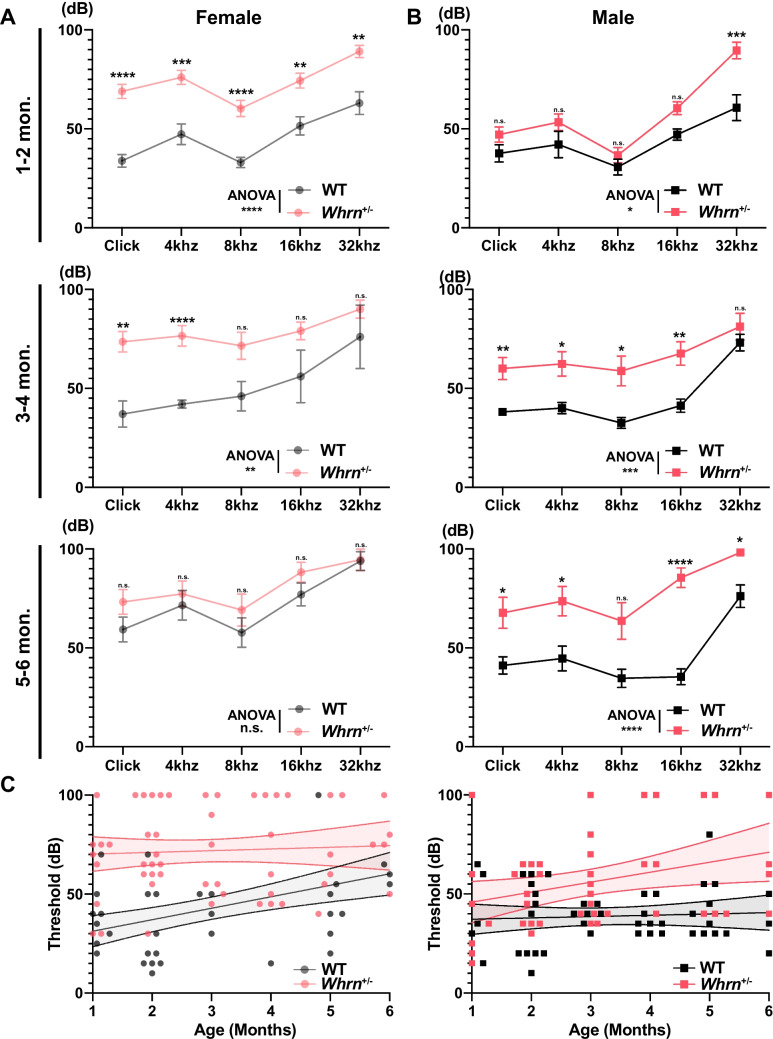
Sexual difference of *Whrn* haploinsufficiency effects. **A**, **B** The threshold of ABRs in responses to click and tone stimuli (4, 8, 16, and 32 kHz) in WT (black) and *Whrn*^+*/–*^ mice at 1–2 months (top, WT = 14–19 males and 20–26 females, *Whrn*^+/–^ = 24 males and 34 females), 3–4 months (middle, WT = 16 males and 5 females, *Whrn*^+/–^ = 17 males and 20 females), and 5–6 months old (bottom, WT = 13 males and 13–14 females, *Whrn*^+/–^ = 11 males and 11 females). Values from female (**A**) and male (**B**) mice are separately displayed. **C** The liner regression plotting of the ABR threshold against different ages (1–6 months old). Dots indicate individual values from females (left) and males (right) in WT (black, *n* = 48 males and 45 females) and *Whrn*^+*/–*^ mice (red, *n* = 44 males and 57 females). Data were shown as mean ± s.e.m., and the regression model was plotted with 95% CI. To test statistical significancy between groups, two-way ANOVA and multiple comparison was used with Šidák correction. The significancy was presented as asterisk (n.s. = not significant, * < 0.05, ** < 0.01, *** < 0.001, **** < 0.0001)

Next, we examined whether sex influences the effect of whirlin haploinsufficiency on hearing sensitivity, because a large variation in ABR threshold within each group was observed (Fig. 1). There was a distinct sexual difference in age-dependent hearing loss between WT and *Whrn*^+/*−*^ mice. *Whrn*^+*/−*^ females exhibited hearing loss in young adulthood, whereas WT females showed age-related hearing loss in this period. In young adulthood females at 1–2 months old, *Whrn*^+*/−*^ females already exhibited hearing loss with a higher threshold than age-matched WT females (two-way ANOVA,* p* < 0.0001; WT, *n* = 20–26; *Whrn*^+*/−*^, *n* = 34). This difference became smaller with age (two-way ANOVA, 3–4 months [WT, *n* = 5; *Whrn*^+*/−*^, *n* = 20],* p* = 0.0072; 5–6 months [WT, *n* = 13–14; *Whrn*^+*/−*^, *n* = 11],* p* = 0.1799), because WT females gradually lose their hearing sensitivity with aging (Fig. 2A).

Interestingly, the pattern of threshold changes in *Whrn*^+*/−*^ males were different from females (Fig. 2B). At 1–2 months old, *Whrn*^+*/−*^ males showed a significantly elevated threshold of ABRs for 32 kHz, indicating high-frequency hearing loss (multiple *t*-test, *p* = 0.0002). The ABR thresholds for other tones in *Whrn*^+*/−*^ males were slightly higher than those from WT males at 1–2 months old (two-way ANOVA,* p* = 0.0156; WT, *n* = 14–19; *Whrn*^+*/−*^, *n* = 24), and the difference in ABR threshold between *Whrn*^+*/−*^ and WT males became significantly increased with aging (two-way ANOVA, 3–4 months [WT, *n* = 16; *Whrn*^+*/−*^, *n* = 17],* p* = 0.0009; 5–6 months [WT, *n* = 13; *Whrn*^+*/−*^, *n* = 11], *p* < 0.0001). *Whrn*^+*/–*^ males have progressive hearing loss indicating the early onset of age-related hearing loss comparing to WT males. To show the relationship between hearing loss and ages in males and females, we performed the linear regression of the change in ABR threshold (Click stimuli) against ages (1–6 months old). The linear regression showed a significant difference in the pattern of threshold change in females and males (Fig. 2C). The slope of linear model and Y-intercept were significantly different in females (*Whrn*^+*/–*^ female: y = 0.853x + 69.41, WT female: *y* = 5.797*x* + 25.49, *p*_slope_ = 0.034). It indicates that *Whrn*^+*/–*^ females have severe hearing loss even as young adults, whereas WT females show age-related hearing loss. For males, although the slope was not different between WT and *Whrn*^+*/–*^ males (*p*_slope_ = 0.086, linear regression), the Y-intercept was significantly different (*p*_intercept_ < 0.0001, linear regression). This indicates that *Whrn*^+*/–*^ males have the early onset of age-related hearing loss compared with WT males showing a normal hearing within the age of 6 months.

### Loss of Outer Hair Cell in *Whrn*^+/−^ Mice

**Fig. 3:**
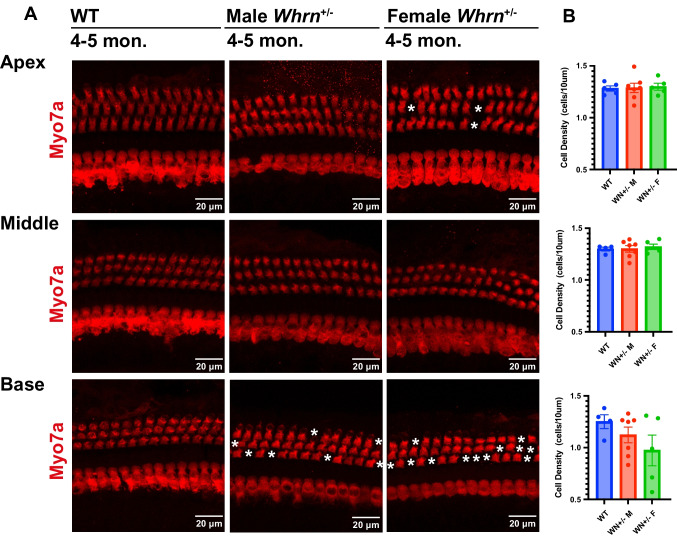
Loss of hair cells in *Whrn*^+*/–*^ mice. **A** Expression Mayo7a in OHCs and IHCs in apical, middle, and basal membrane of the cochlea from *Whrn*^+*/–*^ females (4–5 months old, *n* = 5), males (4–5 months old, *n* = 7), and male WT (4–5 months old, *n* = 4–5) as a control. White asterisks indicate the loss hair cells. **B** The number of OHCs of *Whrn*^+*/–*^ females (green), *Whrn*^+*/–*^ males (red), and WT (blue) at 4–5 months old. Data were shown as mean ± s.e.m. The statistical significance was tested using one-way ANOVA test followed by multiple comparison with Šidák correction, but there was no statistically significant difference between groups

To determine the cellular mechanisms of hearing loss in *Whrn*^+*/–*^ mice, we examined hair cell loss throughout apical to basal regions of the cochlea using immunostaining with antibodies against Myo7a (Fig. 3). The numbers of hair cells were counted at given length (10 μm). The density of OHCs were measured from 3 rows and they were averaged per mouse. The number of OHC in apical (1.282 ± 0.025, *n* = 5 WT mice vs 1.288 ± 0.044, *n* = 7 *Whrn*^+*/–*^ males vs 1.300 ± 0.033, *n* = 5 *Whrn*^+*/–*^ females, Kruskal–Wallis test, *p* = 0.9599) and middle region (1.299 ± 0.019, *n* = 4 WT vs 1.300 ± 0.031, *n* = 7 *Whrn*^+*/–*^ male vs 1.321 ± 0.026, *n* = 5 *Whrn*^+*/–*^ female, Kruskal–Wallis test, *p* = 0.8780) of cochlear were similar between WT and *Whrn*^+*/–*^ mice regardless of sex. The number of OHCs in basal region showed a tendency of reduction (1.251 ± 0.066, *n* = 4 WT vs 1.122 ± 0.076, *n* = 6 *Whrn*^+*/–*^ male vs 0.973 ± 0.148, *n* = 5 *Whrn*^+*/–*^ female, Kruskal–Wallis test, *p* = 0.3869). Although no statistically significant difference was observed in the number of OHCs, there was a clear trend toward a reduced number of OHCs in the basal region of *Whrn*^+*/−*^ mice. These findings suggest that the hearing loss observed in *Whrn*^+*/−*^ mice is not directly attributable to the loss of OHCs, IHCs. However, the observed trend implies that OHCs in the basal region, which are responsible for responding to high-frequency sounds, may be particularly vulnerable to reduced levels of whirlin. Furthermore, the data suggest a potential sex-specific susceptibility, with female mice appearing more vulnerable to the effects of *Whrn* mutations.

## Discussion

Mutations in *Whrn* cause USH2, the most prevalent classification of Usher’s syndrome. The complexity in genotype–phenotype relation of the USH gene, such that mutations in *Whrn* cause various symptoms from moderate sensorineural hearing loss and retinitis pigmentosa to profound sensorineural hearing loss and normal vision, suggests various mechanisms underlying disease manifestations [10]. For studying the mechanisms, several mouse models with *Whrn* mutations are available, including a naturally occurring mutation and targeted mutations in specific regions of the *Whrn* gene. The whirler mouse with *Whrn*^wi^ mutation has a spontaneous 592-bp deletion occurring between exons 6–10, impacting the 3’ region of *Whrn*. Mice with homozygous *Whrn*^wi^ mutations are profoundly deaf consistent with 3’ mutations leading to deafness in DFNB31 patients. In comparison, perturbation of the 5’-region by deleting exon 4 in *Whrn*^tm1b^ mice, disrupts the production of the N-terminal protein and is associated with less severe sensorineural hearing disruptions, similar to those in USH2D [5]. Thus, the severity of the sensorineural hearing loss depends on the location of *Whrn* mutation [5]. Here, we used the *Whrn*^tm1 Tili^ mouse line, where the exon 1 of *Whrn*, the translation start codon for the whirlin long isoform was replaced with Neo expression cassette and disrupt *Whrn* at the 5’-terminal of the *Whrn* transcript [11]. This genetic modification abolishes the long isoform of whirlin without affecting on short isoform, mimicking USH2D phenotypes in humans [11]. Consistently, mice carrying homozygous of *Whrn*^*–/–*^ showed severe hearing impairment at either 2 or 9 months of age [11, 12]. In this study, we found that mice with heterozygous mutation (*Whrn*^+*/–*^) exhibit progressive sensorineural hearing loss in a sex-dependent manner.

USH2 is well known to be inherited as a recessive trait, thus carriers with a single mutated copy of *Whrn* have been considered asymptomatic. Therefore, the potential role for heterozygous mutations in *Whrn* in sensorineural hearing loss has not been evaluated. A recent study on *PDZD7*, another USH2 gene that has high similarity of structure with *Whrn*, suggested the potential haploinsufficiency effect of *PDZD7* on hearing loss. Individuals with de novo heterozygous mutation in *PDZD7* exhibit the early onset of hearing impairment and a stronger retinitis pigmentosa compared to siblings without any mutations in *PDZD7* [4], suggesting the possible impact of *Whrn* haploinsufficiency in USH2 development. In this study, we evaluated the haploinsufficiency effect of *Whrn* on sensorineural hearing loss using *Whrn*^+*/–*^ mice. We found that the loss of a single copy of *Whrn* gene critically impacted the gradual decline of hearing sensitivity and caused progressive hearing loss with tendency of outer hair cell reduction. In addition, we found the sex difference in phenotypes of *Whrn* haploinsufficiency. *Whrn*^+*/–*^ female mice showed a significantly elevated ABR threshold even at 1 month old of age indicating non-progressive hearing defects, whereas WT males showed a gradual increase ABR threshold indicating progressive hearing loss. Notably, the distinct hearing loss in *Whrn*^+*/–*^ females regardless of age shows that females are more susceptible to hearing defects by the loss of a single copy of *Whrn* gene.

In this study, we used *Whrn*^+*/–*^ mice with the C57BL/6 background, which are known to carry mutations in *Cdh23*, encoding cadherin-23, a key component of the tip-link structure in hair cells critical for mechanotransduction. The observed auditory phenotypes and age-dependent variations between male and female mice might be influenced by the presence of the *Cdh23*^ahl^ allele, a hypomorphic variant associated with age-related hearing loss (ARHL). Prior studies on C57BL/6J mice have documented sex differences in hearing thresholds, with females demonstrating higher thresholds than males as they age [13–15]. Our findings align with this observation, suggesting that *Whrn* haploinsufficiency exacerbates hearing loss in a genetic background already predisposed to progressive hearing impairment.

Although Usher syndrome (USH) has traditionally been characterized as a monogenic and genetically heterogeneous disorder, emerging evidence points to digenic inheritance involving interactions between USH genes such as *PDZD7/USH2A* in mice and humans [4, 16–18]. Studies have shown that compound heterozygous mutations in two or more genes can synergistically aggravate sensorineural hearing loss, resulting in severe phenotypes [17, 18]. Similarly, genetic interactions between *Cdh23* and other loci have exacerbated auditory phenotypes. For example, double heterozygosity for mutations in *Cdh23* and *Pcdh15*, another tip-link component, significantly increases susceptibility to hearing loss [19]. A recent study also demonstrated that C57BL/6-*Ush1g*^js/+^ compound heterozygous mice exhibit early-onset progressive hearing loss accompanied by stereocilia degeneration in cochlear OHCs [20]. These findings suggest that the *Cdh23*^ahl^ allele may interact with *Whrn* haploinsufficiency through mechanisms such as increased susceptibility to mechanical stress or impaired stereocilia maintenance and repair.

Interestingly, despite the distinct auditory phenotypes observed in 4–5-month-old *Whrn*^+*/–*^ mice, there was no significant loss of OHCs or inner hair cells IHCs in the cochlea in the current study. We hypothesize that this early-onset progressive hearing loss is initiated by stereocilia defects, followed by OHC loss at later stages (9–12 months), as shown by Miyasaka et al. [20]. Moreover, previous studies indicate that the progressive hearing loss phenotype in *Whrn*^+*/–*^ may be recovered by correcting the strain-specific *Cdh23*^ahl^ mutation [20, 21]. This suggests that the interplay between *Cdh23*^ahl^ and *Whrn* mutations is a significant factor in the observed phenotype.

Our results also revealed sex-specific differences in auditory phenotypes among *Whrn*^+*/–*^ mice. Although the mechanisms underlying the sexual dimorphism in *Cdh23*-related hearing loss in C57BL/6 mice remain unclear, it is plausible that compound heterozygous mutations in *Whrn* and *Cdh23* act synergistically. Further research is required to elucidate these mechanisms and determine how genetic background and sex-specific factors influence auditory outcomes in this model.

In summary, our study provides novel insights into the complex interactions between *Whrn* haploinsufficiency and the *Cdh23*^ahl^ allele in progressive hearing loss. Future studies incorporating targeted genetic manipulations and detailed phenotypic assessments are necessary to further unravel the molecular mechanisms underlying these interactions and their contributions to hearing loss.

## Data Availability

All data has been presented in the manuscript.
